# NblA1/A2-Dependent Homeostasis of Amino Acid Pools during Nitrogen Starvation in *Synechocystis* sp. PCC 6803

**DOI:** 10.3390/metabo4030517

**Published:** 2014-06-30

**Authors:** Hiroshi Kiyota, Masami Yokota Hirai, Masahiko Ikeuchi

**Affiliations:** 1 Department of Biological Sciences, Graduate School of Science, The University of Tokyo, 7-3-1 Hongo, Bunkyo-ku, Tokyo 113-0033, Japan; E-Mail: kiyo_hiro@bio.c.u-tokyo.ac.jp; 2 RIKEN Center for Sustainable Resource Science, 1-7-22 Suehiro-cho, Tsurumi-ku, Yokohama, Kanagawa 230-0045, Japan; E-Mail: masami.hirai@riken.jp; 3 Department of Life Sciences (Biology), Graduate School of Arts and Science, The University of Tokyo, 3-8-1 Komaba, Meguro-ku, Tokyo 153-8902, Japan

**Keywords:** cyanobacteria, nbl, amino acid, nitrogen metabolism, *Synechocystis*

## Abstract

Nutrient balance is important for photosynthetic growth and biomass production in microalgae. Here, we investigated and compared metabolic responses of amino acid pools to nitrogen and sulfur starvation in a unicellular model cyanobacterium, *Synechocystis* sp. PCC 6803, and its mutant *nblA1/A2*. It is known that NblA1/A2-dependent and -independent breakdown of abundant photosynthetic phycobiliproteins and other cellular proteins supply nutrients to the organism. However, the contribution of the NblA1/A2-dependent nutrient supply to amino acid pool homeostasis has not been studied. Our study demonstrates that changes in the pool size of many amino acids during nitrogen starvation can be categorized as NblA1/A2-dependent (Gln, Glu, glutathione, Gly, Ile, Leu, Met, Phe, Pro, Ser, Thr, Tyr and Val) and NblA1/A2-independent (Ala, Asn, Lys, and Trp). We also report unique changes in amino acid pool sizes during sulfur starvation in wild type and the mutant and found a generally marked increase in the Lys pool in cyanobacteria during nutrient starvation. In conclusion, the NblA1/A2-dependent protein turnover contributes to the maintenance of many amino acid pools during nitrogen starvation.

## 1. Introduction

Nutrient balance is critical for the phototrophic growth of microalgae, because proteins, nucleic acids, carbohydrates, lipids, and pigments must be supplied in a ratio suitable for actively growing cells. Once the nutrient supply is out of balance, microalgae try to accommodate by inducing nutrient uptake, reducing photosynthesis, suppressing growth and autophagy, *etc.* [1,2]. In contrast, it is often important to limit nutrient supply, e.g., when microalgae produce certain biomass compounds [3]. For commercial purposes, it is desirable to artificially separate biomass production from cell proliferation in microalgae, which allows the microalgae to be used as a photobioreactor for the production of a specific biomass. Gene expression during nutrient starvation and related stresses has been studied extensively in microalgae using DNA microarray and RNA sequencing (RNA-Seq) analyses [4,5]. Little, however, is known about the precise mechanisms underlying metabolic acclimation to nutrient starvation.

Nitrogen is a key macronutrient for microalgae, which adjust for nitrogen availability by global or specific regulation of gene expression, metabolic pathways, protein turnover, photosynthetic capacity, and the cell cycle [6,7]. In eukaryotic microalgae, autophagy is induced by nitrogen starvation to degrade cytoplasmic components including plastids in the large vacuoles [8]. In cyanobacteria, a unique NblA-dependent mechanism is induced to degrade certain phycobiliproteins, which are major components of the phycobilisome, the supramolecular photosynthetic antenna that harvests light for photosynthesis in these organisms. They are soluble proteins attached to the thylakoid surface and may constitute up to ~50% of total soluble proteins in many cyanobacterial cells [9]. Upon nitrogen—and sometimes sulfur—starvation, cyanobacteria degrade most of these blue-colored phycobiliproteins, resulting in a bleached cell color. Phycobiliprotein degradation has been believed to contribute to the supply of macronutrients that are necessary to maintain cellular functions during nutrient starvation [10]. The non-bleaching phenotype gene (*nblA*) was identified as an essential factor for the specific degradation of phycobiliproteins [11,12]. Because the small protein NblA directly binds to certain phycobiliproteins, its binding may trigger proteolytic breakdown of the target proteins [13]. It is, however, still unknown to what extent NblA-dependent protein degradation contributes to the amino acid supply during nitrogen starvation [14,15,16,17].

The unicellular cyanobacterium *Synechocystis* sp. PCC 6803 (hereafter *Synechocystis*) is a model species for basic and applied research of photosynthesis, metabolism, and many other cellular processes. The complete genome of the standard strain PCC 6803 was determined in 1996, and many related substrains have been sequenced recently [18]. DNA microarray, RNA-Seq, proteome, and metabolome analyses of this organism have been carried out in many laboratories [19,20]. Regulation of photosynthetic metabolisms has been studied by metabolic phenotyping under stress conditions (high light and low CO_2_) and trophic conditions (photoautotrophic and photomixotrophic) [21,22]. More recently, this species has also been used for metabolic engineering and biomass production [23,24,25]. *Synechocystis* does not fix molecular nitrogen but assimilates ammonium, nitrate, and other compounds into amino acids. Upon nitrogen starvation, the global nitrogen regulator NtcA mainly regulates genes for carbon and nitrogen metabolism [26,27]. Expression of *nblA*, which consists of two tandemly arranged *nblA1* and *nblA2* loci in *Synechocystis*, is induced under starvation for nitrogen [28]. The phycobilisome is believed to have a role in nitrogen storage as well as its role as a photosynthetic antenna, but this hypothesis has not yet been tested [29].

To investigate the contribution of amino acid recycling in *Synechocystis*, we therefore analyzed the metabolic responses of amino acid pools in *Synechocystis* and its mutant *nblA1/A2* to nitrogen and sulfur starvation. 

## 2. Results and Discussion

### 2.1. Responses of Amino Acid Pools to Nitrogen Starvation in Synechocystis Cells

**Figure 1:**
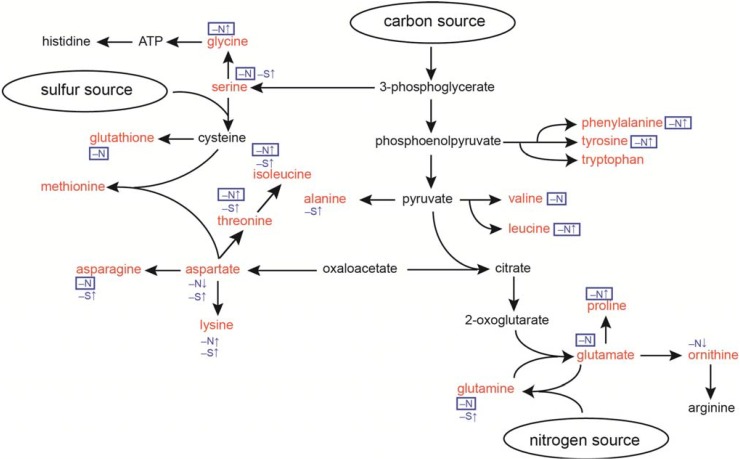
Amino acid biosynthesis pathways in *Synechocystis* and summary of responses during nitrogen or sulfur starvation. Amino acids shown in red were analyzed in this study. Responses to nitrogen starvation (–N↑ or –N↓) or to sulfur starvation (–S↑) are indicated in blue and the responses that depend on NblA1/A2 are boxed. Note that –N stands for amino acids, whose levels are maintained by NblA1/A2 during nitrogen starvation.

**Figure 2:**
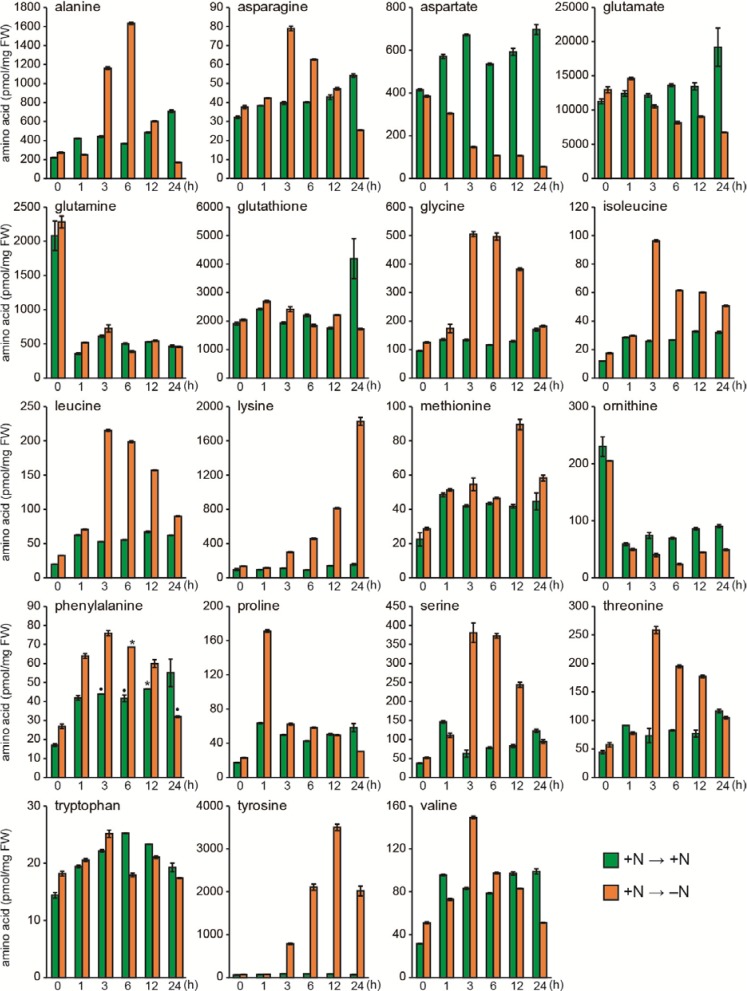
Changes in amino acid pools of cells during nitrogen starvation. Orange bars: cells cultured in nitrogen free medium (0 ~ 24 h), green bars: control cells cultured in the nitrate containing medium (0 ~ 24 h) after the same medium exchange treatment as the nitrogen free cells. Note that cells at 0 h were collected just after the medium exchange treatment. Standard deviation is shown from three measurements (n = 3), but asterisks and dots above bars in phenylalanine represent n = 2 and n = 1, respectively.

To quickly and quantitatively extract free amino acids from *Synechocystis* cells, cells were collected with a filter and disrupted with zirconia beads, and free amino acids were then extracted with methanol. The free amino acids were derivatized and subjected to gas chromatography–mass spectrometry (GC-MS) analysis as described [30]. This method provided quantitative data for free amino acids, except Arg and His, as depicted in the biosynthesis pathway (Figure 1). Figure 2 shows the change in the free amino acid pools in *Synechocystis* cells during 24-h nitrogen starvation. Note that Cys levels were not reported, because the Cys pool size was below the detection limit (<0.2 pmol/mg fresh weight [FW]). We also quantified non-standard amino acids, ornithine and glutathione. Ornithine is an intermediate of Arg biosynthesis. Glutathione is a redox carrier consisting of Cys, Glu and Gly (Figure 1).

In the presence of sufficient nitrogen, the most abundant amino acid was Glu, followed by glutathione (2000–12,000 pmol/mg FW). The pool size of Ala, Asp, and Gln was high (100–300 pmol/mg FW), whereas the remaining amino acid pools were low in abundance. This free amino acid profile is consistent with previous reports in *Synechocystis* [19], the unicellular cyanobacterium *Anacystis nidulans* [15], and the multicellular cyanobacterium *Arthrospira platensis* [14]. Similar patterns are also found in *Escherichia coli* and *Saccharomyces cerevisiae* [31,32]. In contrast, patterns in higher plants, such as *Arabidopsis thaliana*, are quite different [33]. When *Synechocystis* cells were collected by filtration, suspended, and cultured in N-free medium for 24 h under otherwise normal culture conditions, we found two types of responses (Figure 2). The first is a transient increase after 1–5 h, followed by a return to the starting levels (Ala, Asn, Gly, Ile, Leu, Met, Phe, Pro, Ser, Thr, and Val). The second is a long-term increase throughout the 24-h incubation period (Lys and Tyr). In contrast, we found a severe decline in Asp. The pool size of Asp, Glu, and ornithine gradually declined to approximately half of the control level and remained low for 24 h. It should be noted that a marked increase of Gln and ornithine was found only at 0 h (just after washing) irrespective of whether the N-deficient or N-containing medium was used for washing. Therefore, this is likely an effect caused by the medium change, whereas the other responses most likely result from changes in amino acid biosynthesis, supply from protein degradation, and interconversion among amino acids and other metabolites during nitrogen starvation. The overall responses of amino acid pools during nitrogen starvation were similar to those reported in cyanobacteria, although some variation in the time course and/or extent of responses occurred [14,15,16,17]. We did not study further responses beyond 24 h but the long-term responses would also be important for regulation and maintenance of metabolites. Hauf *et al.* [17] reported some differential responses in *Synechocystis* cells after 168 h of nitrogen starvation.

### 2.2. Responses to Nitrogen Repletion following Starvation

**Figure 3:**
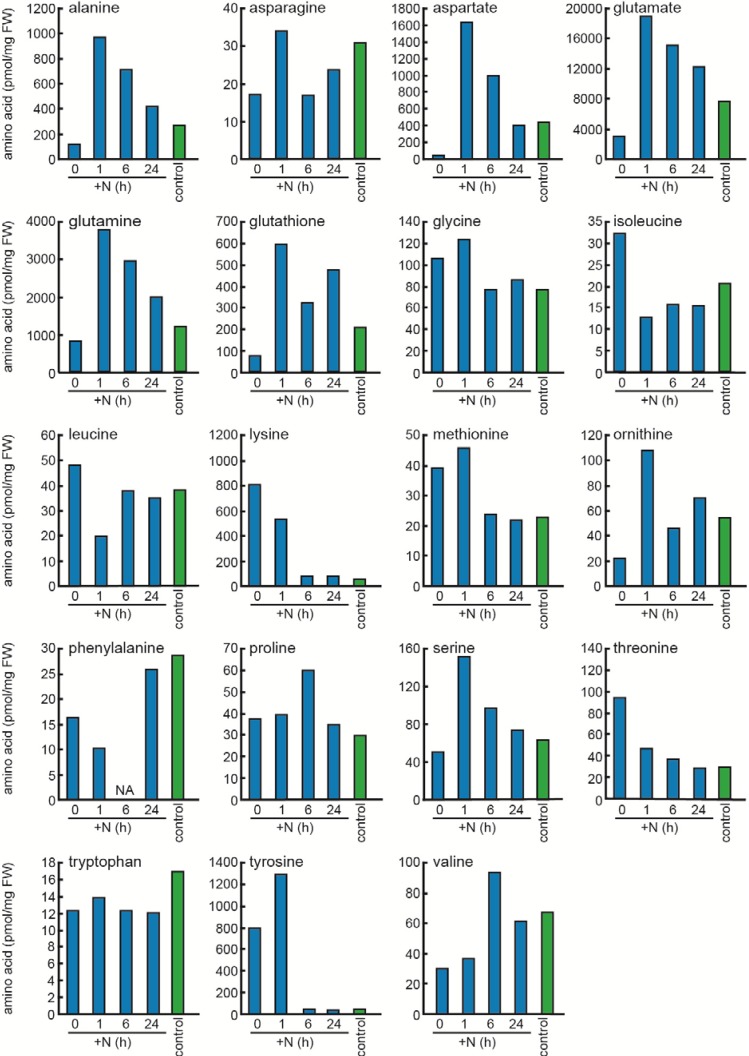
Effects of nitrogen re-addition on the amino acid pools of *Synechocystis* cells that were nitrogen starved for 24 h. Blue bars: cells nitrogen starved for 24 h (0 h), or then cultured after re-addition of nitrate (1, 6, 24 h). Green bars: control cells grown under nitrogen containing condition. NA, not assigned in gas chromatography (GC).

Next, we investigated the effects of nitrate re-addition on the amino acid pools of cells that have been nitrogen starved for 24 h (Figure 3). The long-term changes in Asp, Glu, Lys, ornithine, and Tyr during nitrogen starvation were approximately reversed by the subsequent addition of nitrogen. Thus, we are able to confirm the reversible responses of amino acids upon nitrogen starvation and nitrogen re-assimilation, although the experiment was done once. More specifically, the pool size of Lys and Tyr did not change much after 1 h but fully recovered to its original level 6 h after re-addition. The long-term accumulation of Lys and Tyr during starvation may be due to the suppression of *de novo* protein biosynthesis and the degradation of cellular proteins, whereas the reversion to the original level during the repletion may result from the recovery of protein biosynthesis. The pool size of Asp, Glu, and ornithine transiently overshot and then dropped to levels found in nitrogen-sufficient conditions. A similar overshoot was found in Ala, Gln, and Ser. The reversion of the overshoot could be due to the recovery of protein biosynthesis, whereas the overshoot itself could result from nitrogen re-assimilation in the absence of protein biosynthesis, because these amino acids can be readily produced from the nitrogen source via central metabolites (Figure 1).

### 2.3. Response of the nblA1/A2 Mutant to Nitrogen Starvation

**Figure 4:**
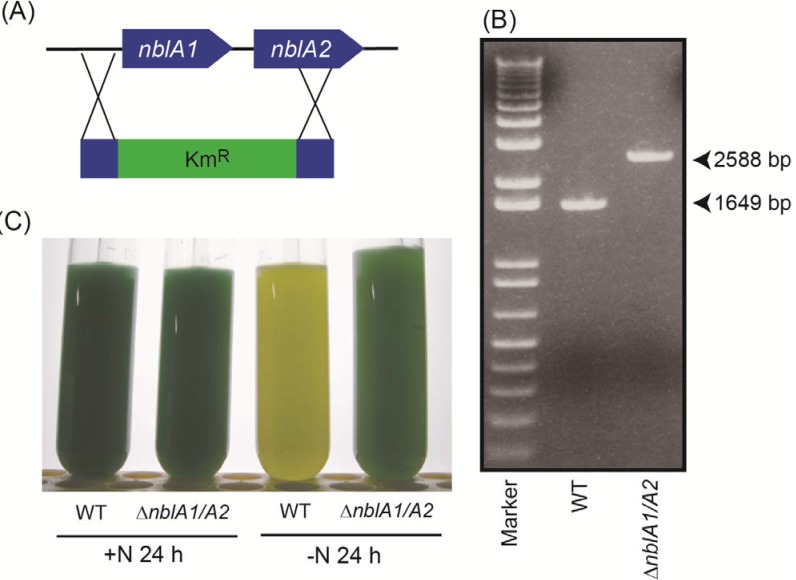
(**a**) Deletion of *nblA1/A2* by a kanamycin resistance cassette (Km^R^); (**b**) PCR analysis of *nblA1/A2*. The calculated DNA size of each PCR product is 2588 bp for the *nblA1/A2* mutant (∆*nblA1/A2*) and 1649 bp for wild type; (**c**) Non-bleaching phenotype of the *nblA1/A2* mutant under nitrogen starvation. WT, wild type.

**Figure 5:**
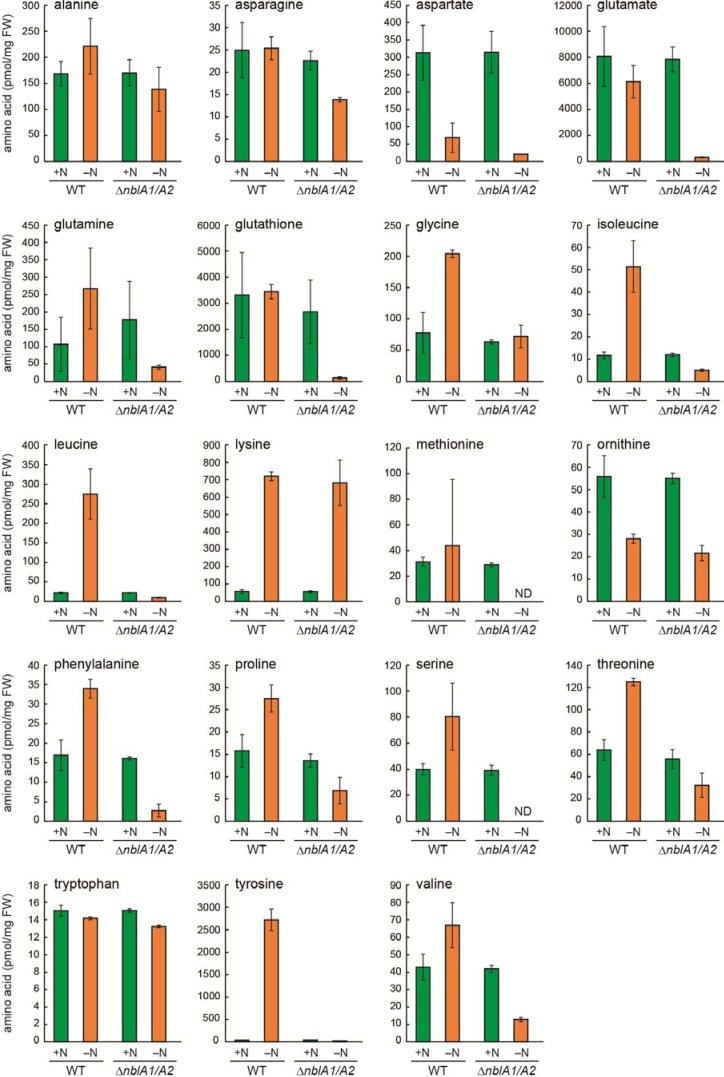
Amino acid pools in WT *Synechocystis* and the *nblA1/A2* mutant (∆*nblA1/A2*) after 24 h in nitrogen-sufficient (+N) or nitrogen-deficient (–N) medium. Standard deviation is shown from three independent experiments. ND, not detected.

To study the turnover of phycobiliproteins, we examined the pool size of free amino acids in the *nblA1/A2* mutant during nitrogen starvation. First, the tandemly arranged *nblA1* and *nblA2* were replaced with the screening cassette (Figure 4A), and complete segregation was confirmed by PCR (Figure 4B). We also visually confirmed the non-bleaching phenotype of the mutant during nitrogen starvation (Figure 4C). Then, the pool size of free amino acids was analyzed after 24 h of nitrogen starvation. The increase of Gly, Ile, Leu, Phe, Pro, Thr and Tyr was abolished in the *nblA1/A2* mutant, whereas the increase of Lys was not affected at all (Figure 5). The decrease of Asp and ornithine was not affected, either. For the remaining amino acids, their pool sizes were maintained after 24 h of nitrogen starvation, irrespective of the presence or absence of transient changes. These were divided into two groups: NblA1/A2-dependent amino acids and NblA1/A2-independent ones. The NblA1/A2-dependent group includes Asn, Gln, Glu, Ser, Val, and glutathione. The NblA1/A2-independent group includes Ala, and Trp. It should be noted that the NblA1/A2-dependent nitrogen supply was critical for Glu and glutathione homeostasis in cells during nitrogen starvation.

**Figure 6:**
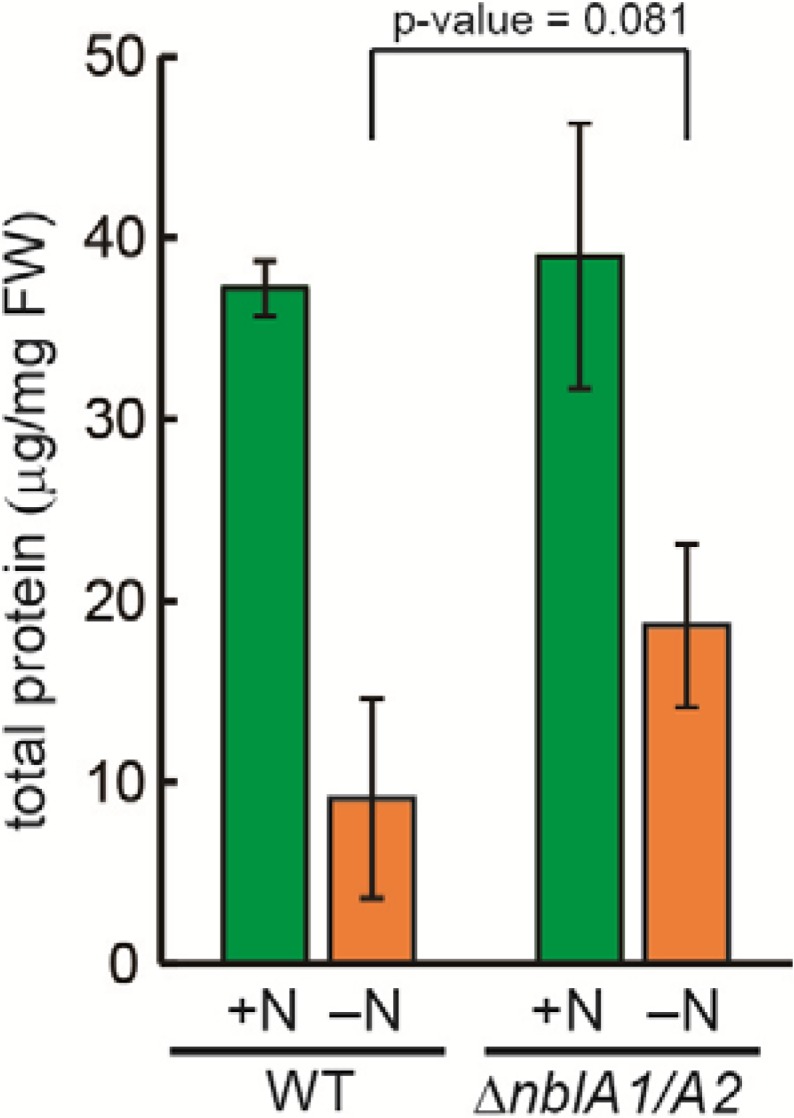
Total amount of cellular protein in WT *Synechocystis* and the *nblA1/A2* mutant (∆*nblA1/A2*) after 24 h in nitrogen-sufficient (+N) or nitrogen-deficient (–N) medium. Standard deviation is shown from three independent experiments.

To evaluate the overall contribution of NblA1/A2 to amino acid pool sizes, the total protein content of wild-type and mutant *Synechocystis* cells was compared. In wild-type cells, the protein content (relative to the fresh cell weight) was reduced to one-fourth during 24 h of nitrogen starvation (Figure 6). In the mutant, protein content before starvation was comparable to that of the wild type, whereas it was reduced to nearly one-half of the initial level after starvation. This suggests that approximately one-third of the protein turnover triggered by nitrogen starvation depends on the NblA1/A2-induced breakdown of the abundant phycobiliproteins, whereas the remaining two-thirds of protein turnover may be due to the breakdown of other proteins.

Nitrogen supply in the NblA1/A2-dependent group of amino acid pools must be derived from the major phycobilisome components, which consist of two phycobiliproteins, CpcA and CpcB, and two colorless linker proteins, CpcC1 and CpcC2. During nitrogen starvation, it is generally believed that two outer hexameric discs of phycobiliproteins are degraded together with each associated linker protein, although the extent of degradation depends on the severity and duration of the starvation. The amino acid composition of these proteins is reasonably similar to the NblA1/A2-dependent group noted above. In this context, it is important to note that the NblA1/A2-dependent amino acids are found in a relatively large amount in the CpcA/B proteins, though the independent ones such as Ala, Asp, and Lys are also contained. It is thus suggested that the latter amino acids may also be supplied from other sources.

### 2.4. Response of the nblA1/A2 Mutant to Sulfur Starvation

**Figure 7:**
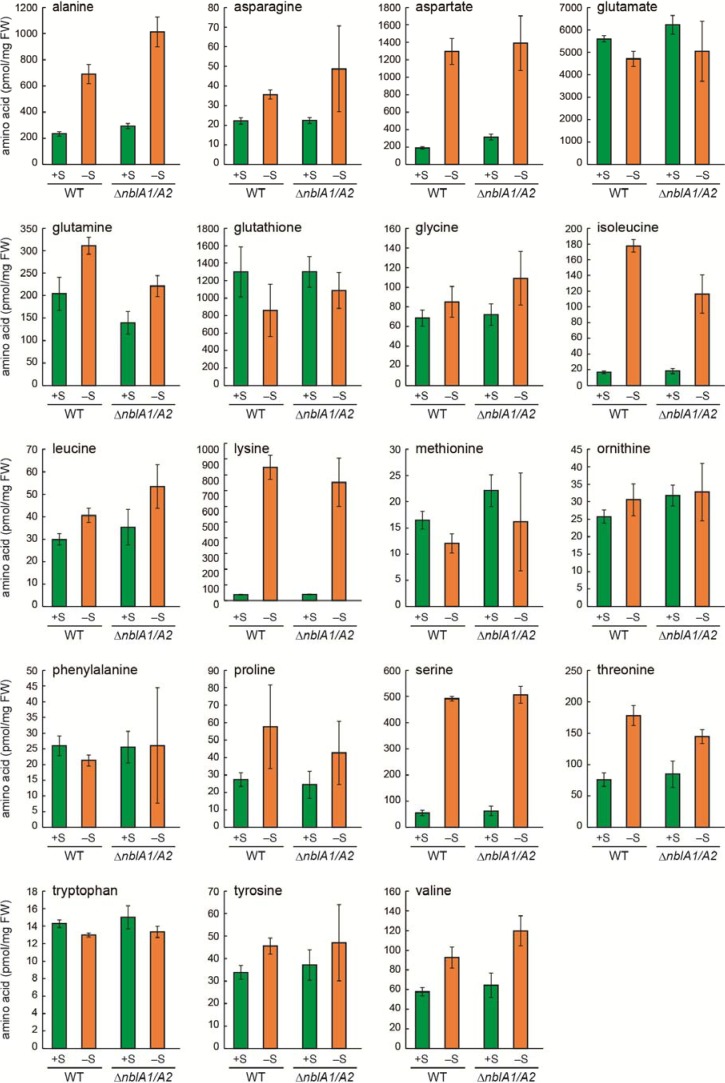
Amino acid pools in WT *Synechocystis* and the *nblA1/A2* mutant (∆*nblA1/A2*) after 24 h in sulfur-sufficient (+S) or sulfur-deficient (–S) medium. Standard deviation is shown from three independent experiments.

We also examined amino acid pools of the *nblA1/A2* mutant under sulfur starvation (Figure 7). In wild-type cells, Ala, Asn, Asp, Gln, Ile, Lys, Ser, and Thr increased 24 h after starvation, in agreement with our previous report [30]. None of these amino acids were affected by the disruption of *nblA1/A2*. This is consistent with a report that sulfur starvation does not induce phycobilisome degradation in *Synechocystis* [34].

### 2.5. NblA1/A2–Dependent and –Independent Homeostasis of Amino Acids

In Figure 1, we summarized the NblA1/A2-dependent and -independent amino acid homeostasis during nitrogen and sulfur starvation in the model cyanobacterium *Synechocystis* sp. PCC 6803. The amino acids, which responded or were maintained during nitrogen starvation by NblA1/A2, are highlighted with a box, whereas the NblA1/A2-independent ones, which responded either to nitrogen or sulfur starvation, are also indicated but not highlighted. Thus, we can conclude that many amino acids (Asn, Gln, Glu, glutathione, Gly, Ile, Leu, Phe, Pro, Ser, Thr, Tyr, and Val) can be at least in part supplied from phycobilisome components via NblA1/A2–mediated rapid turnover. We can also assume that sulfur starvation suppressed conversion of Asp–to–Met and Ser–to–Cys, which may lead to accumulation of Asp, Ser and other amino acids nearby (Figure 1), as previously reported [24]. However, the enhanced accumulation of Lys is not correlated with the pool size of Asp (high during sulfur starvation, but low during nitrogen starvation). This finding may rule out the previous interpretation for Lys accumulation via Asp during sulfur starvation. There are no Lys–metabolizing enzymes annotated for *Synechocystis* in the Kyoto Encyclopedia of Genes and Genomes (KEGG) pathway [35]. Moreover, there are many Lys–rich proteins hypothetically coded for in the *Synechocystis* genome [18]. Turnover of such proteins and the absence of Lys-metabolizing enzymes might result in Lys accumulation during nitrogen and sulfur starvation. On the other hand, the increase of Ala and Gln was unique to sulfur starvation, whereas the decrease of Asp and ornithine was unique to nitrogen starvation irrespective of NblA1/A2 protein. Thus, some selective acclimation might also occur in amino acid homeostasis to mitigate each nutrient starvation. Further characterization in the common and specific acclimation in amino acid recycling would give a clue for metabolic plasticity of phototrophic organisms.

## 3. Experimental Section

### 3.1. Culture Conditions and Cell Sampling

Cells of *Synechocystis* sp. PCC 6803 glucose-tolerant substrain [17] were grown under normal growth conditions at 30 °C in BG11 medium supplemented with 20 mM HEPES-KOH (pH 7.8) [36] with bubbling 1% (v/v) CO_2_ and continuous illumination with white fluorescent lamps (30–40 μmol∙s^−1^∙m^−2^). For medium changes, cells at an optical density (*A*_730_) of 1.0–1.5 were harvested by filtration and resuspended in fresh BG11 medium, nitrogen-free BG11(–N) medium, or sulfur-free BG11(–S) medium. In BG11(–N), 17 mM NaNO_3_ was replaced by 17 mM of NaCl. In BG11(–S), 0.3 mM MgSO_4_ was replaced by 0.3 mM MgCl_2_. After 24 h, cells were collected by filtration and the cell pellet scraped were weighted, frozen in liquid nitrogen, and preserved at −80 °C. For experiments of nitrogen re-addition, 1.5 mM NaNO_3_ was simply added to the nitrogen-starved cells.

### 3.2. Disruption of nblA1/nblA2

A DNA fragment harboring *nblA1* and *nblA2* was amplified from genomic DNA by PCR using the primers ACAACATTGGGCACGAGA and ACTACCATGATCAATCCC. The fragment was then cloned into a pT7Blue vector. The entire region of *nblA1* and a portion of *nblA2* were excised by *Mfe*I and *Hpa*I and replaced with a kanamycin resistance cassette. *Synechocystis* cells were transformed by homologous recombination and screened in the presence of 20 µg/mL kanamycin. Complete segregation of the mutant allele was confirmed by PCR using the same primers as mentioned above.

### 3.3. Extraction of Amino Acids

To disrupt the cells, 1 mL of 60% (v/v) methanol in H_2_O and 1 g of zirconia beads were added to the frozen cell pellet at 4 °C, followed by vortexing twice for 30 s with a 1-min interval between vortexing. The mixture was centrifuged at 9000 g for 5 min, and the supernatant was collected. The beads were washed with 500 µL of 60% methanol, centrifuged at 20,000 g for 5 min, and the supernatant was collected. Combined extracts were dried and stored until further analysis. [30].

### 3.4. Derivatization and GC-MS Analysis of Amino Acids

Dried extracts were dissolved in 60% (v/v) methanol containing 2 nmol of norvaline as an internal standard and derivatized using EZ:faast™ (Phenomenex, Torrance, CA, USA). GC-MS was performed using the QP2010 Plus GC-MS (Shimadzu, Kyoto, Japan) equipped with a ZB-AAA column (Phenomenex). The analytical conditions were as follows: carrier gas, He (1.17 mL/min); ionization voltage, 70 kV; injector temperature, 280 °C; and oven program, 110 °C (1-min hold), with an increase of 20 °C min^−1^ to 300 °C (2-min hold). Analyses were carried out in Selected Ion Monitoring mode. Each amino acid was identified and quantified in the extracted ion chromatogram at its respective retention time. Quantified data were normalized to the fresh weight of the frozen cell pellet [30].

### 3.5. Bradford Assay

To extract total cellular proteins, we added zirconia beads and a protein extraction buffer (100 mM NaCl, 10% (w/v) glycerol, 20 mM HEPES-NaOH (pH 7.5)) to the frozen cells and vortexed the mixture at 3000 rpm for 30 sec. After centrifugation at 10,000 g, the protein content of the supernatant was detected using a Bradford kit [30].

## 4. Conclusions

We studied the effects of protein turnover on pool sizes of free amino acids in cyanobacteria, including in the *nblA1/A2* mutant, during nitrogen and sulfur starvation. The abundant phycobiliproteins are degraded specifically during nitrogen starvation in an NblA1/A2-dependent manner. Many other cellular proteins are also degraded to supply free amino acids during such nutrient starvation. The contribution of the NblA1/A2-dependent supply to nutrient starvation, however, has not been studied. Here we demonstrated that the changes in pool size of many amino acids during nitrogen starvation can be categorized into NblA1/A2-dependent (Gln, Glu, glutathione, Gly, Ile, Leu, Met, Phe, Pro, Ser, Thr, Tyr and Val) and NblA1/A2-independent (Ala, Asn, Lys, and Trp). We confirmed that there are changes in amino acid pools that differ under nitrogen and under sulfur starvation and found that the marked increase in the Lys pool is a general event that occurs in cyanobacteria during nutrient starvation. The mechanism and biological role of this increase in Lys remain unknown but its marked accumulation may be adopted for some applied purpose in a photosynthetic biomass production.
